# Inkjet-Printed Rough Gold Microelectrode Arrays on Flexible Substrates for Neural Recording and Electrical Stimulation

**DOI:** 10.1021/acsami.6c01300

**Published:** 2026-05-04

**Authors:** Amelie Ziller, Andrea Corna, Mai Thu Bui, Paul Werginz, Michael Schneider, Ulrich Schmid, Günther Zeck

**Affiliations:** † Institute of Biomedical Electronics, 27259 TU Wien, Gußhausstraße 27-29, 1040 Vienna, Austria; ‡ Institute of Sensor and Actuator Systems, 27259 TU Wien, Gußhausstraße 27-29, 1040 Vienna, Austria

**Keywords:** microelectrode arrays, additive manufacturing, neuroelectronics, extracellular recording, electrical stimulation, ex vivo retina

## Abstract

Microelectrode arrays (MEAs) are essential tools for recording and stimulating electrogenic tissues, but their fabrication typically depends on complex, costly, and mask-based cleanroom processes. While inkjet-printed MEAs have increasingly been explored as low-cost alternatives, most demonstrations have focused on cardiac cell recordings, with only a limited number of studies showing neuronal recordings. Furthermore, no work to date has demonstrated neuronal interfacing, combining single-unit recording with electrical stimulation, using inkjet-printed MEAs. Here, we investigate whether inkjet-printed MEAs enable both extracellular single-unit neuronal recording and reliable electrical stimulation. We fabricated gold microelectrodes on flexible foils via maskless inkjet-printing, insulated them with printed SU-8 (an epoxy-based dielectric), and characterized their morphology using scanning electron microscopy, atomic force microscopy, and profilometry, and their electrochemical behavior using impedance spectroscopy and cyclic voltammetry. The printed gold formed a rough nanoparticle-based morphology, resulting in an increased effective electrochemical surface area. This morphology enabled low electrode impedances and high charge injection during voltage-controlled stimulation. We assessed functional performance in ex vivo retinal tissue. The inkjet-printed MEAs enabled reliable single-unit recordings with signal-to-noise ratios comparable to cleanroom-fabricated commercial devices and cell activation upon electrical stimulation with biphasic pulses. The electrodes were reusable and noncytotoxic, verified via a standard cell viability assay. These results establish the first inkjet-printed microelectrodes capable of neuronal interfacing, demonstrating that printed MEAs can match the functional performance of conventional microfabricated devices. This work positions inkjet-printing as a scalable, easily adaptable, low-cost manufacturing technique for flexible MEAs with rough gold electrodes suitable for neurotechnology applications.

## Graphical abstract

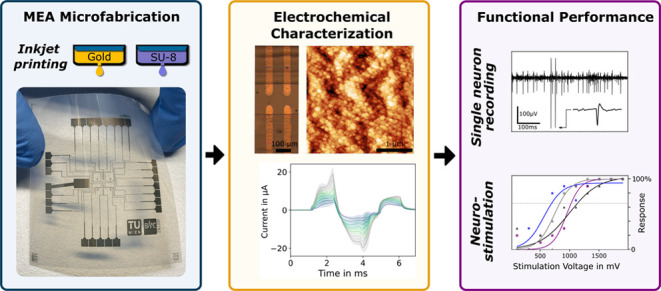

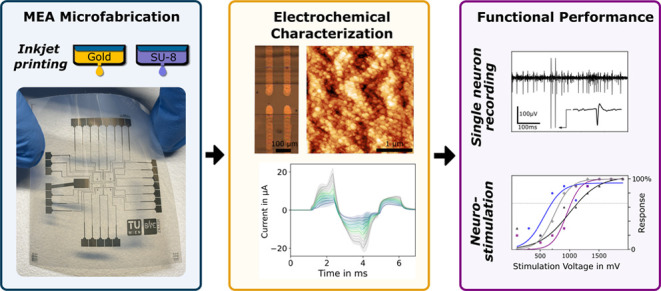

## Introduction

Microelectrode arrays (MEAs) are versatile platforms that enable precise electrical recording and stimulation of neural or cardiac tissues, making them essential tools for interfacing electrogenic systems and for studying how these networks respond to modulatory inputs. Their applications range from basic neuroscience and drug testing to neuroprosthetics, and they are used across multiple models, from in vivo cortical recordings to organoids and in vitro preparations.^1,2^ Yet most standard MEA manufacturing relies on cleanroom processes that are costly, slow, and difficult to adapt for flexible substrates or small-batch customization. We therefore propose a maskless inkjet-printing approach that enables fast, low-cost, and adaptable fabrication of MEAs on flexible substrates. Through electrochemical and functional characterization in biological models, we demonstrate that our inkjet-printed MEAs achieve performance comparable and, in part superior, to devices manufactured in a cleanroom.

Inkjet-printing is a noncontact, scalable, material-efficient deposition technique allowing for the fabrication of conductive and of insulating structures with feature sizes as small as 40–50 μm making it well-suited for manufacturing MEAs. The process utilizes low-viscosity inks to additively deposit materials. The materials can be water-based conductive metal nanoparticle inks, which can be sintered at temperatures as low as 60 °C, allowing the production of noncytotoxic electrodes on heat-sensitive substrates. Combined with its compatibility with a wide variety of materials, ranging from rigid substrates to flexible foils and thin, delicate films, inkjet-printing enables the fabrication of mechanically compliant devices that better match the properties of soft biological tissues,^3^ while offering a short design-to-fabrication cycle that greatly benefits rapid prototyping in research and development.

Overall, the combination of fast processing, a broad selection of conductive inks, and high patterning flexibility provides a powerful, easily adaptable fabrication platform for MEAs intended for in vitro, ex vivo, or wearable electronic applications, including neural recording and stimulation.

Previous studies have explored inkjet-printed electrode arrays for cardiac muscle cell recording using carbon, gold, poly­(3,4-ethylenedioxythiophene):poly­(styrenesulfonate) (PEDOT:PSS), and graphene inks. For instance, Adly et al. reported silk carbon electrodes printed for recordings from HL-1 cardiac muscle cells, while Bachmann et al. employed a custom-made gold nanoparticle ink, Garma et al. utilized PEDOT:PSS, and Lumpuy-Castillo et al. inkjet-printed graphene ink, also targeting HL-1 cells.^4–7^ More complex 3D structures were introduced by Grob et al., using inkjet-printed silver pillars electroplated with gold for HL-1 cells, and later extended to cortical organoids by Kopic et al.^8–10^ Further approaches included screen-printed carbon electrodes for retinal stimulation and recording,^11^ and in vivo neural recordings with inkjet-printed PEDOT:PSS with gold, and platinum electrodes.^12,13^ While these studies demonstrate the feasibility of printed MEAs with biological samples, the proof of these platforms with neuronal interfacing is still limited. Most prior work is restricted to HL-1 cardiac recordings, with only a few demonstrations of neuronal recordings, in organoids^8,9^ and in vivo^12,13^ with large electrocorticography electrodes and with 4 electrode optrodes. Only one screen-printed platform has targeted retinal tissue,^11^ with modest signal-to-noise ratios (SNRs). To our knowledge, no prior work has demonstrated extracellular recording and stimulation of single-unit neuronal activity using inkjet-printed electrodes.

Here, we present the first inkjet-printed MEA on a flexible substrate featuring single-cell-level neuronal recording and electrical stimulation capabilities. The gold electrode arrays insulated with printed epoxy-based negative photoresist SU-8 fabricated on flexible polyethylene-naphthalate (PEN) foils were characterized morphologically by scanning electron microscopy (SEM), atomic force microscopy (AFM), by thickness profilometry, and electrochemically using cyclic voltammetry and impedance spectroscopy. Functionally, we demonstrate neuron recording and stimulation in ex vivo retinal preparations, achieving SNRs, cathodic charge densities and single-spike resolution superior to commercially produced, cleanroom-fabricated gold-MEAs. Stimulation thresholds were within the same operational range as previously reported values. The electrodes are reusable and their noncytotoxicity was confirmed by cell counting kit-8 (CCK-8) assay. Taken together, these results position inkjet-printed MEAs as a scalable, cost-effective platform for flexible neural interfaces suitable for in vitro, ex vivo, and wearable applications.

## Results and Discussion

### Fabrication of Printed MEAs

Printed electrode arrays were fabricated by inkjet-printing on PEN foils. The overall manufacturing process is shown in Figure 1a. Electrode arrays were fabricated in four steps: (1) printing of electrodes and feedlines with gold ink; (2) curing of the printed gold at 80 °C; (3) printing of SU-8 as the insulating layer; and (4) postprocessing of the SU-8.

**Figure 1:**
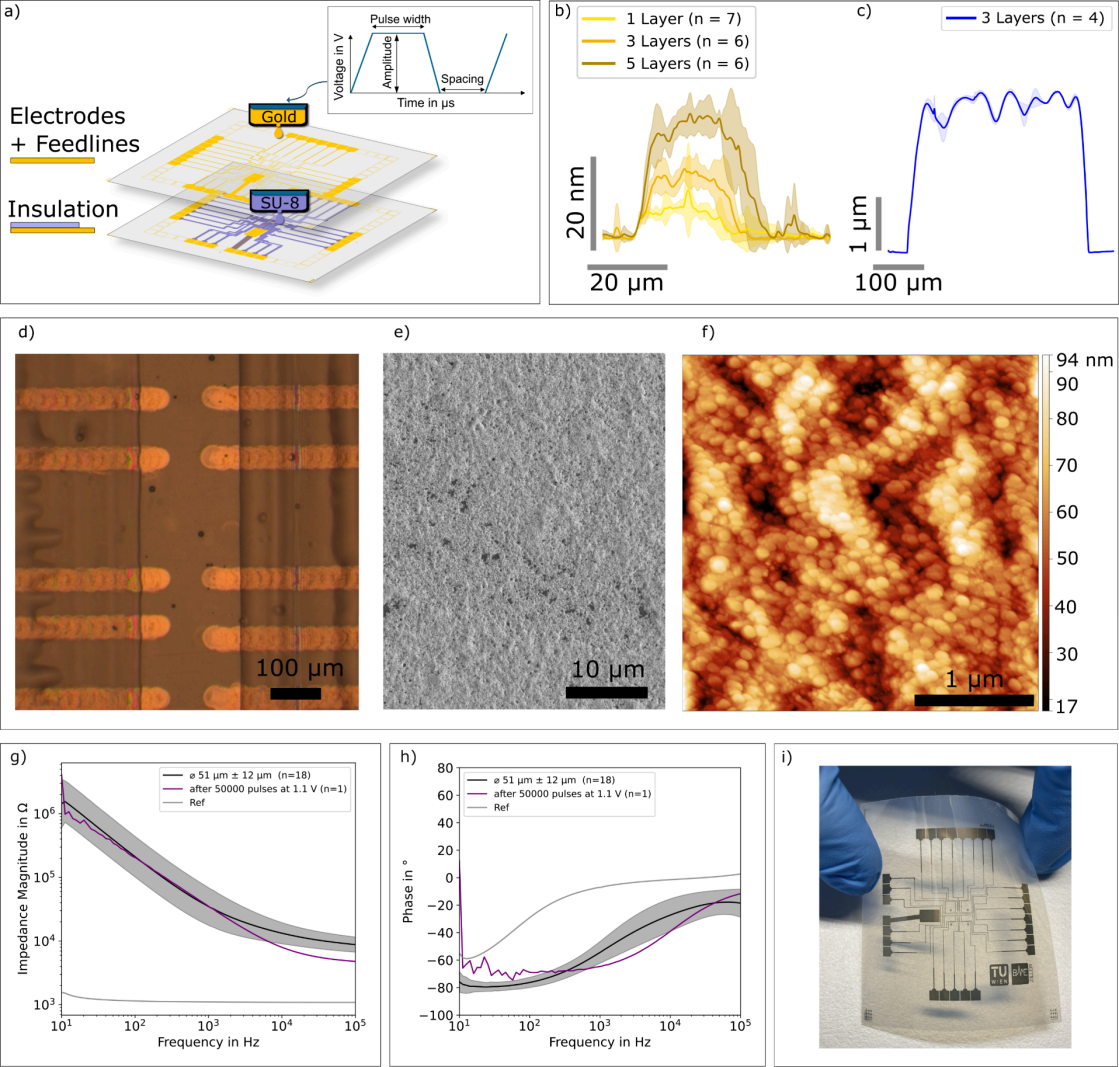
Fabrication and characterization of the inkjet-printed electrode array with rough gold surfaces. (a) Fabrication of printed electrode arrays by material deposition via piezo-based inkjet-printing. (b) Average height profile and standard deviation of a printed gold line on glass for 1, 3 and 5 print passes. (c) Printed SU-8 insulates a gold trace of poly­(methyl methacrylate) (PMMA) substrate, (d) brightfield microscopy image of ten electrodes and complete flexible electrode array overview, (e) scanning electron microscopy image of the printed gold (3 layers) on polyethylene-naphthalate (PEN), (f) atomic force microscopy image of gold (5 layers) on PEN. The image area is 3 × 3 μm^2^. (g) Average impedance magnitude and standard deviation of printed electrodes (*n* = 18), along with the impedance spectrum obtained after 50,000 pulses (1.1 V, 5 ms pulses with 500 ms break between pulses). (h) Average phase and standard deviation (*n* = 18). (i) Ready-to-use flexible printed MEA.

Before printing conductive and insulating structures, we analyzed and optimized the droplet behavior of both inks. For SU-8, the ink was diluted based on surface tension and viscosity measurements. A dilution with 11 wt % cyclopentanone yielded surface tension and viscosity values within the recommended range for the printing cartridge, whereas lower cyclopentanone concentrations increased viscosity and higher concentrations decreased it (see Printing Technology and Ink Preparation section; Figure S1a–c). The gold ink was printed as purchased.

A printing speed of 300 mm s^–1^ and a density of 1200 × 1200 dots per inch (dpi), defining drop spacing in the *x*- and *y*-directions, produced continuous gold and SU-8 structures. At lower dpi values, printed lines became discontinuous, while at higher dpi values, the line width increased, potentially causing short circuits between adjacent feedlines. To avoid micron-scale misalignments, short circuits, and open feedlines, which occurred when printing in both directions or when using multiple nozzles, we printed here in a single direction using a single nozzle.

### Geometrical Characterization of the Printed MEAs

To evaluate whether inkjet-printing can reliably produce microelectrodes suitable for neuronal recording and stimulation, we first characterized the dimensions and surface properties of the printed electrodes. In inkjet-printing, pixels of the digital design are translated into individual ink droplets deposited on the substrate. The minimum achievable droplet diameter determines the spatial resolution of the printing process. Electrodes were defined by an area of 2 × 3 pixels which was not covered by SU-8. Adjacent sensing sites were separated by a pitch of 4 pixels in the *y*-direction and 5 pixels in the *x*-direction. This yielded electrodes with an equivalent average diameter of 51 μm (*n* = 18, SD = 11.6 μm) and an electrode pitch of 117 μm in *y* (*n* = 8, SD = 11.2 μm) and 142 μm in *x*-direction (*n* = 6, SD = 7.2 μm) (Figure 1d,i).

AFM analysis revealed a root-mean-square surface roughness of *r*_q_ = 16.1 nm (*n* = 3, SD = 3.9 nm) (Figure 1f) for the printed gold. For comparison, photolithographically patterned gold exhibits a roughness of about 2 nm.^14^ Surface profilometry further demonstrated that the printed gold could be deposited in well-defined, repeatable multilayers: single-, triple-, and five-layer gold prints exhibited proportional increases in thickness, with the five-layer structures reaching ∼25 nm, confirming that layer stacking can be achieved with good vertical accuracy and without significant spreading or feature distortion. This multilayer capability is important for tuning electrode thickness and conductivity while maintaining geometric precision. Conducting feedlines encapsulated with printed SU-8 on poly­(methyl methacrylate) (PMMA) showed an insulation thickness of ∼3 μm, sufficient to fully cover the nanometer-thin gold traces and prevent electrolyte access during operation. Device-to-device impedance variability is low, exact values are listed in Table S1 and optical images are shown in Figure S2. Despite limitations inherent to inkjet-printing, such as droplet formation dynamics, motor precision, and substrate wetting behavior, the resulting electrode dimensions fall within the established range for functional microelectrodes (<50–70 μm) suitable for extracellular recording and neural stimulation.^15^ These findings demonstrate that inkjet-printing can reproducibly produce electrode sizes, pitches, and surface profiles compatible with high-quality neural interfacing.

### Electrochemical Characterization of the Printed Electrodes

To assess whether the printed electrodes provide suitable performance for electrophysiological recordings, we characterized their electrochemical behavior using electrochemical impedance spectroscopy (EIS). A low impedance magnitude for microelectrodes is usually aimed for as this allows the detection of small extracellular voltages. Since the thermal noise contribution at the electrode increases with increasing impedance, EIS gives important insight into the electrode’s capability to record small signals at high SNRs.^16^ Electrode impedance magnitude also depends on electrode area. Consequently, smaller electrodes typically exhibit higher impedance because of their reduced electrochemically active area.^16,17^

The magnitude and phase of the electrochemical impedance of the printed electrodes were measured and analyzed (Figure 1g,h). Impedance magnitudes decreased with increasing frequency. Gold electrodes with an average diameter of 51 ± 12 μm had an average impedance magnitude of ∼41 kΩ at 1 kHz. Despite their relatively small geometric size, these values are comparable to previously reported larger gold microelectrodes,^5,18^ and lower than the impedance magnitudes of sputtered gold electrodes with similar diameters reported in literature^17^ (Table 1). This result can be explained by the 8-fold increase in surface roughness and thus a higher effective electrochemical surface area. The phase shift increased toward higher frequencies, indicating a resistive behavior. The phase at 1 kHz was −56° (Figure 1h), which is comparable to previous work,^4^ and more capacitive than other reported phase values.^11^ The cutoff frequency at a phase angle of −45° (*f*_cutoff_) was calculated to provide information on the filtering characteristics of the electrodes. The gold electrodes presented here showed an average *f*_cutoff_ of 3.1 kHz, similar to previous values.^4^ The impedance magnitude and the phase do not change after appling 50,000 pulses (amplitude 1.1 V, frequency: 1.98 Hz) and after sterilization with UV light, ethanol cleaning, coating with poly-l-lysin and multiple retina experiments (see purple trace in Figure 1g,h). Futher evaluation of the impedance upon stimulation with higher amplitudes is presented and discussed in the section “Neurostimulation with Printed MEAs”.

**Table 1:** Impedance Magnitude Values at 1 kHz with Their Corresponding Electrode Diameters

**electrode type**	**printed (this work)**	**printed**	**sputtered**	**sputtered**	**sputtered (commercial)**
impedance (kΩ at 1 kH)	41	20	36	100	558/141
diameter (μm)	51 ± 12	103 ± 27	200	50	40/110

Overall, the electrochemical measurements show that the printed electrodes achieve impedance characteristics suitable for high-quality electrophysiological recordings.

### Specific Resistivity of Printed Gold Feedlines

The specific resistivities of the printed conductive lines were measured to ensure sufficient conductivity along the feedlines. We printed nine gold lines with contact pads on glass substrates and measured each line’s resistance three times using a wafer prober. Line thickness was measured two to three times using a height profilometer. For each line, we averaged the cross-sectional area and calculated the specific resistivity individually. The printed gold feedlines showed an average specific resistivity of 19 ± 3 μΩ cm (*n* = 9). These specific resistivity values fall within the range reported for a similar noncommercial gold ink formulated by Bachmann et al. (18–76 μΩ cm, sintered at 125 °C^5^), confirming that the resistivity achieved with our printing and curing conditions is consistent with previous work.

### Recording Neural Activity with the Printed Electrodes

To evaluate whether printed electrodes detect neuronal extracellular action potentials with amplitudes in the range of few hundreds of μV and durations around 1 ms, an ex vivo mouse retina was interfaced. The electrodes were positioned in close contact with the retinal ganglion cell (RGC) layer. The experimental setup and a representative micrograph are shown in Figure 2a. We recorded spontaneous extracellular action potentials (“spikes”) in the explanted mouse retina over extended durations and identified single spikes on multiple electrodes of the same MEA (Figure 2b). The signals were band-pass filtered between 200 and 5000 Hz (second-order Bessel). Spike amplitudes reached up to 350 μV, with an average SNR of 5.3, comparable to previously reported values obtained using photolithographically fabricated MEAs in ex vivo retina (6.5;^19^ which is higher than the SNR estimated on screen-printed MEAs with ex vivo retina,^11^ and in agreement with typical SNRs reported by others (≥5 ^20^). The SNR should be at 5 to 1 or higher for reliable recording of extracellular action potentials with spikes in the order of 100 μV.^15^ Thus, the printed electrodes achieve SNRs sufficient for robust spike detection.

**Figure 2:**
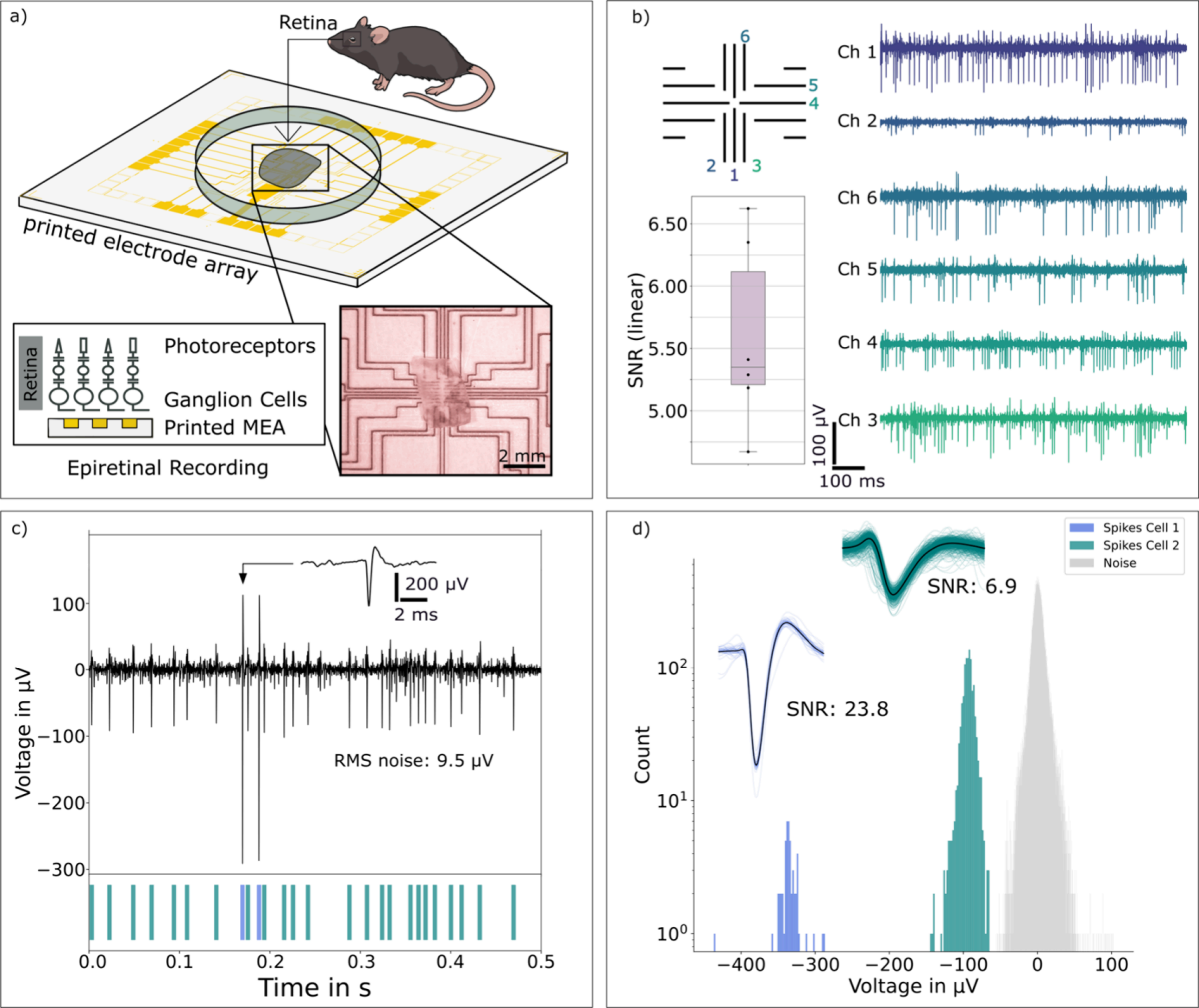
Low-noise neural recording and spike sorting with the printed electrode array. (a) Setup used for ex vivo epiretinal mouse retina recording. Inset shows brightfield image of the printed electrodes with the interfaced ex vivo mouse retina. (b) Filtered extracellular voltage recordings from several electrodes of one printed electrode array. The signal-to-noise ratios (SNRs) are shown in boxplot. The spike amplitudes are visualized here in the range of +60/–110 μV. (c) Recording of spiking activity within 500 ms with a magnified single spike and the color-coded results after spike sorting. Spikes of Ch1 (Figure 2b) are shown here, as well as the root mean square (RMS) noise. (d) Histogram of extracellular voltages sorted with a clustering algorithm into two cells and noise. The mean waveforms and their SNRs of the two identified units are displayed.

Recordings from individual MEAs were stable for more than 1 h and were reproducible across multiple experimental sessions over the course of 6 months, indicating long-term electrode stability. The longest recording lasted 5 h with stable spike amplitudes and spike rates (Figure S3). The recording duration is comparable to previously reported values for passive MEAs^20^ and CMOS MEAs^21,22^ using the same type of ex vivo retina.

Spike sorting based on principal component analysis (PCA) of waveform features enabled assignment of extracellular signals recorded by a single electrode to different cells (Figure 2c). The histogram in Figure 2d shows the spike count over 30 s, with a bin width of 2 μV (bin size of 187) for spikes and bin size of 1000 for noise, demonstrating clear separation of spikes from noise. For other electrodes in Figure 2b, the spike sorting may give less conclusive results, as is the case for other recording approaches.^23^ In summary, as demonstrated here for acute recordings in retinal neural tissue, the printed electrodes are well-suited for multiple, long-term (≥5 h) electrophysiological recordings at high resolution and high SNR.

### Neurostimulation with Printed MEAs

Printed MEAs offer improved neurostimulation capabilities, because of the increased effective electrochemical surface area resulting from the printed gold nanoparticles. To investigate the stimulation performance of the printed electrodes and select an appropriate stimulus waveform, we applied voltage-controlled pulses and compared biphasic square-wave pulses (Figure 3a) with biphasic ramps (Figure 3b) and recorded the resulting current using a transimpedance amplifier. Voltage-controlled stimulation was chosen to directly monitor the electrode response during stimulation.

**Figure 3:**
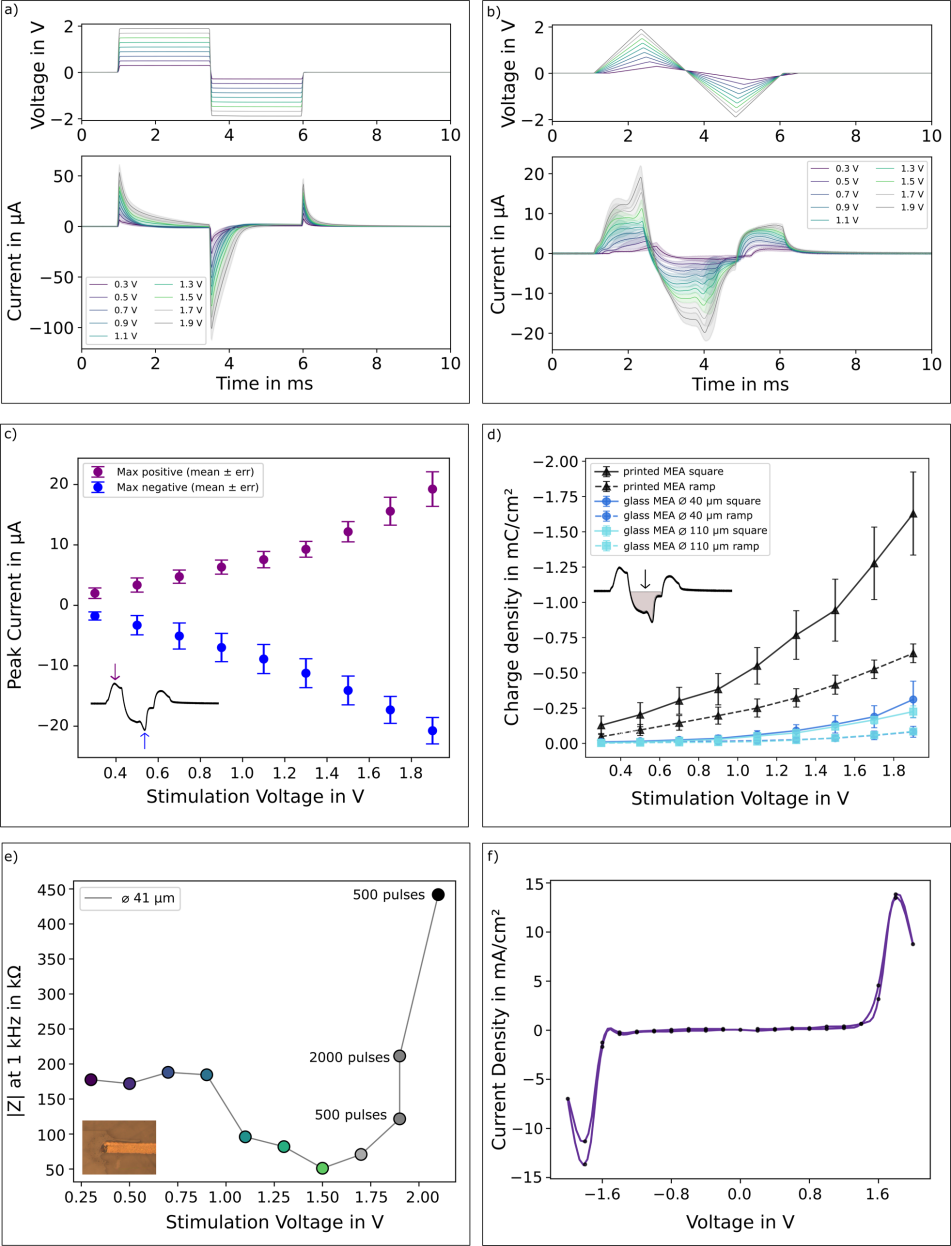
Electrochemical characterization of electrical stimulation with printed microelectrodes, leading to high cathodic charge densities. (a) Average stimulation current (*n* = 3 single electrodes) with standard deviations (*n* = 20 repetitions) at different voltages upon square voltage-controlled pulse. (b) Average stimulation current as in (a) for ramp-shaped voltage-controlled pulse with pauses between pulses of 500 μs. (c) Cathodic (purple) and anodic (blue) peak currents at the applied voltages for ramp-shaped pulses. (d) Cathodic charge density in mC cm^–^^2^ corresponding to stimulation voltages for square and ramp pulses. Printed microelectrodes were compared to sputtered gold microelectrodes. (e) Impedance magnitude after continued stimulation evaluated at 1 kHz. (f) Current density recorded with cyclic voltammetry (0.1 V s^–1^).

Voltage ramps were evaluated because, for purely capacitive stimulation, this waveform generates approximately constant current pulses.^18^ Such currents can be more easily subtracted from the recorded extracellular signal, enabling improved separation of stimulation artifacts and neural activity. In addition, the injected charge is distributed more evenly over the duration of the pulse rather than being concentrated at the initial voltage transition.

As shown in Figure 3b–d, the maximum stimulation current and the cathodic charge density increased with the applied voltage up to 1.9 V. The resulting stimulation current reflects both capacitive and faradaic contributions at the electrode–electrolyte interface. The current is primarily governed by capacitive charging, while the subsequent decay is determined by the RC time constant of the system. At higher voltages, a second rise of current was observed, indicating increasing faradaic contributions. This behavior arises because once the applied voltage exceeds a certain threshold, the gold surface interacts with ions in the electrolyte, leading to processes such as gold oxide formation and interactions with chloride ions. These reactions introduce additional faradaic currents that contribute to the overall current response, as shown in Figure 3b. The electrochemical behavior of gold electrodes in electrolyte has been reported previously.^18,24^ For ramp-shaped pulses, the negative maximum stimulation current reached ∼20 μA at 1.9 V, corresponding to a cathodic charge density of −0.64 mC cm^–2^. For sputtered gold electrodes, similar current waveforms were observed. However, both the peak currents and the cathodic charge densities were substantially lower, reaching −0.08 mC cm^–2^ for 40 μm electrodes as well as for 110 μm electrodes. This enhanced stimulation performance is attributed to the increased effective surface area of the rough printed gold electrodes.

To assess the stability of the electrodes under electrical stimulation, we evaluated the electrode impedance magnitude after pulsing. Sets of 500 biphasic voltage ramp-shaped pulses were applied at fixed amplitudes, starting at 0.3 V and subsequently increasing up to 1.9 V. For pulse amplitudes ≤1.5 V, the 1 kHz impedance magnitude consistently decreased, consistent with electrochemical surface cleaning and removal of adsorbates^25,26^ and the results of the cyclic voltammetry (CV) in Figure 3f. After an additional 1500 pulses at 1.9 V, the impedance magnitude remained stable, indicating that the electrodes tolerated extended pulsing at this amplitude without measurable degradation. In contrast, applying 500 pulses at 2.1 V amplitude caused a sharp impedance magnitude increase (Figure 3e), indicating the onset of irreversible oxidation and corrosion at the electrode.^24,27^ From these measurements, a safe charge injection limit of approximately −0.6 mC cm^–2^ can be estimated for the applied ramp pulses, although this limit is expected to depend on pulse shape and duration and may vary under current-controlled or chronic stimulation conditions.

After contact with biological samples, a slight increase in impedance magnitude was observed (Figure 3e, impedance magnitude at 0.3 V), likely due to adsorption of biological material on the electrode surface. This effect was reversible, as electrochemical cleaning using CV restored the impedance magnitude to ∼50 kΩ at 1 kHz.

After establishing the favorable electrochemical performance of the printed gold microelectrodes over sputtered gold microelectrodes, electrical stimulation of the retina was performed. These experiments were conducted using ex vivo retina in a “sandwich configuration,” where the photoreceptor side contacted the printed MEA and the RGC layer interfaced with a second, flexible, perforated MEA (Figure 4a). A similar configuration to study neurostimulation with a silicon implant has been employed previously by our group.^28^ Here, we extend this approach for the first time to printed electrode arrays on flexible substrates. This configuration enabled stable stimulation and recording of neuronal activity for at least 1 h.

**Figure 4:**
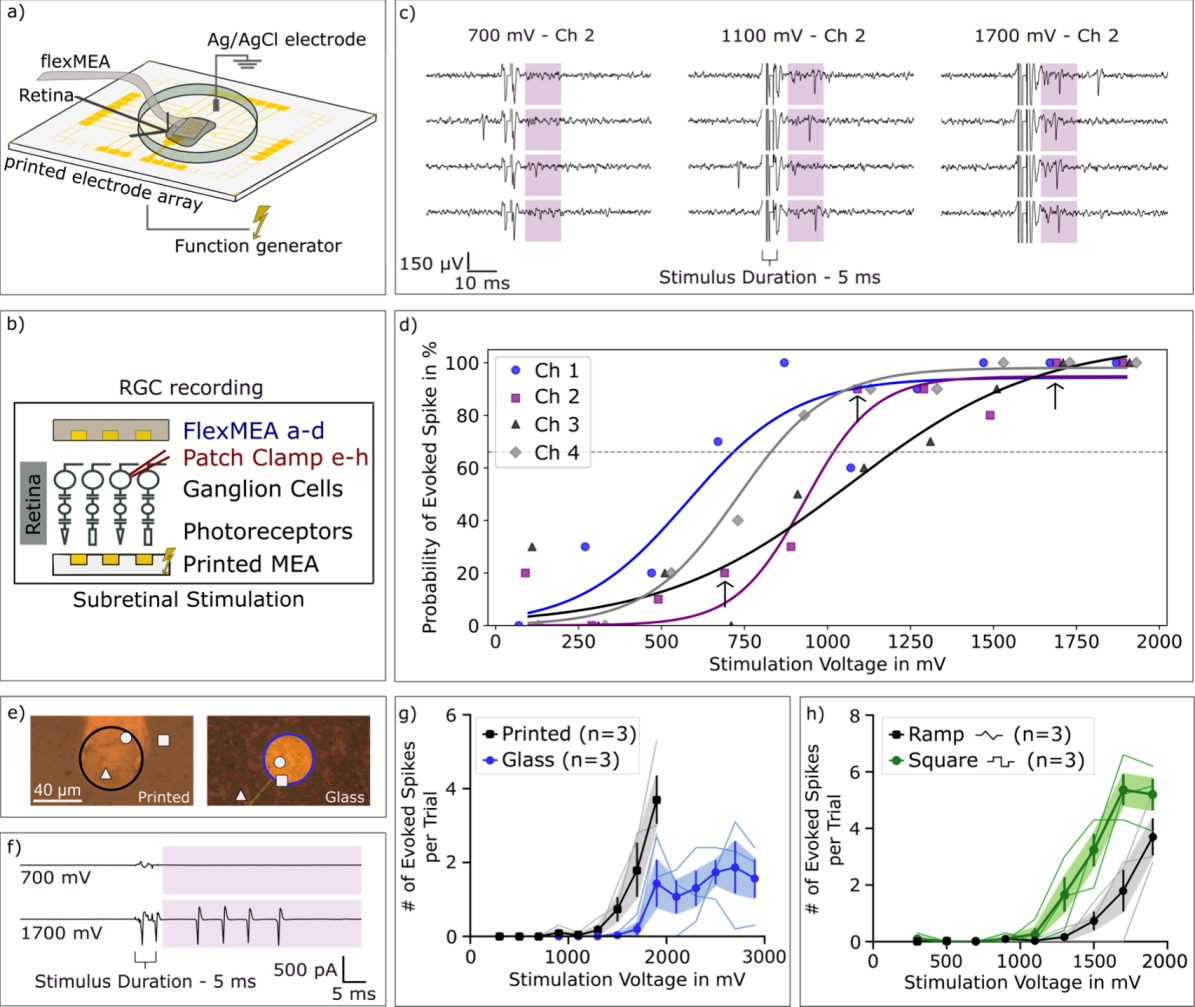
Electrical stimulation of retinal neurons is improved on printed electrodes. (a) Setup for the retinal stimulation. (b) Cross-section of the recording and stimulation setup. (c) Neural activation at 700, 1100 and 1700 mV stimulation, and the shaded response window. (d) Probability to evoke a spike measured for four different stimulation electrodes (Ch1–4) in one retina with fitted sigmoidal curves. The arrows show the measurements depicted in (c). (e) Location of recorded retinal ganglion cells (RGCs) relative to the stimulating printed microelectrode array (MEA) (left) or sputtered gold microelectrode on glass (glass MEA) (right). (f) Representative responses from a RGC stimulated by the printed MEA. While stimulation at 700 mV resulted in no response stimulation at higher levels led to robust spiking responses. (g) Number of spikes over stimulation amplitude is plotted for the printed MEA (black) versus the photolithographically manufactured glass MEA (blue). (h) Number of spikes over stimulation amplitude is plotted for ramp stimulation (black) versus square pulse stimulation (green) for the printed MEA. Thick lines and colored shadings in (g) and (h) show mean ± standard error of the mean.

Successful stimulation of neural tissue mainly relies on the delivery of sufficient charge within a defined time window. For retinal neuroprosthetic applications a stimulus duration of 1–5 ms is usually employed and a fast neural response within 10–40 ms that may allow for continuous light perception is desired.^28–31^ In this study, 5 ms biphasic ramp-shaped voltage pulses were applied.

To evaluate the stimulation performance of the printed MEAs, these pulses (Figure 3b) were delivered to the interfaced retina while recording the neural activity. The recorded voltage traces are shown in Figure 4c. Repetition of the identical stimulation pulse evoked neuronal activation, confirmed by the detected extracellular spikes. The probability of evoking a spike was tested for four electrodes in one retina, testing each electrode separately and sequentially (Figure 4d). The stimulation thresholds, i.e. the voltage amplitude where the sigmoidal fit crosses 66% of the maximum response were 691 mV (ch1), 1021 mV (ch2), 1130 mV (ch3) and 828 mV (ch4) respectively. We infer an average threshold of 917 mV, which corresponds to a cathodic charge density threshold of approximately −0.20 mC cm^–2^. This threshold value is as high as inferred previously for subretinal stimulation using implantable iridium oxide electrodes.^28,32^ All thresholds (<1.2 V) remained within the safe stimulation regime. The variability of individual threshold values may arise from distance differences between the stimulation electrode and the stimulated cell.

The flexMEA recordings allowed us to demonstrate the feasability of RGC activation using the printed MEA. In a next set of experiments, we sought to investigate RGC responses in more detail using patch clamp electrophysiology which allowed us to precisely track the location of the recorded cell relative to the stimulating electrode as well as count spikes during stimulation. We recorded 3 RGCs stimulated by the printed MEA (Figure 4e, left) and 3 RGCs stimulated by a commercially available glass MEA (Figure 4e, right). Both MEAs were able to reliably activate target cells (Figure 4f, printed MEA) with thresholds in the range of 1.5 V, slightly higher than thresholds measured in the sandwich configuration (Figure 4d). While stimulation using both MEAs resulted in similar activation thresholds, stimulation with the printed MEA led to a higher number of action potentials for a given amplitude (Figure 4h) indicating more efficient stimulation paralleling the higher charge injection of the printed MEA shown in Figure 3d. We also compared stimulation with biphasic voltage ramps versus stimulation with biphasic square pulses which demonstrate higher efficiency for square pulses in line with higher peak current and higher charge injection (see Figure 3c,d). Square voltage pulses are typically not used in neuroprosthetic applications such as artificial vision. Instead, nearly constant current pulses are employed to encode light perception.^33,34^ Here, voltage ramps were employed to approximate this behavior, enabling charge modulation via pulse width.

While electrode stability was not the primary focus of this study, these results define an operational window for printed gold electrodes and point to future material optimization possibilities. Compared to sputtered gold electrodes, the inkjet-printed electrodes exhibit increased effective surface roughness, lower impedance magnitude, and higher cathodic charge density, enabling more efficient electrical stimulation. Overall, these results demonstrate that the printed microelectrode arrays can deliver physiologically relevant stimulation currents with predictable and stable charge injection characteristics and reliably evoke neuronal responses in ex vivo mouse retina. Thus, we demonstrate a fully printed interface capable of both neuronal recording and stimulation.

Compared to cleanroom-fabricated MEAs, these additively manufactured MEAs offer advantages in adaptability, rapid prototyping, cost and material reduction, and compatibility with novel substrates. Future efforts will focus on improving charge injection capacity and long-term stability through electrodeposition of advanced materials like PEDOT:PSS on top of the gold electrodes and on scaling and miniaturizing these devices for high-density, flexible bidirectional neurointerfaces.^35,36^

### Cytotoxicity Tests of the Printed Materials

Cytocompatibility of the printed materials was assessed over a 7 day culture period using the CCK-8 assay to evaluate whether inks deposited on PEN are suitable for direct cell contact. CCK-8 is a colorimetric viability assay that detects dehydrogenase activity in viable cells via reduction of water-soluble tetrazolium salt-8 (WST-8), producing a soluble orange formazan quantified at 450 nm (Figure 5a).^37,38^

Across all time points, SU-8 and gold, coated with collagen to facilitate cell adhesion, consistently supported high cell viability. On day 1, viability was comparable to the reference, collagen-coated PEN, reaching 101.03% on SU-8 and 101.96% on gold. By day 2, SU-8 and gold maintained viability at 87.48 and 73.05%, respectively, with no statistically significant differences from the control. Similar trends were observed on day 3 (80.57% on SU-8, 75.45% on gold) and day 7 (88.24% on SU-8, 86.59% on gold) (Figure 5b).

In contrast, poly-l-lysine (PLL)-coated surfaces, included as negative controls^39^ exhibited the expected progressive loss of viability, declining to 49.94% (day 2), 25.63% (day 3), and 32.08% (day 7) on PEN, with similar reductions on SU-8 and gold (Figure 5b). These results demonstrate that the assay detects low viability under the experimental workflow, supporting the interpretation that the high viability on collagen-coated printed inks reflects their cytocompatibility. Overall, these findings suggest that all printed materials are noncytotoxic and support sustained cell viability, indicating their suitability for long-term cell–material interfacing..

**Figure 5:**
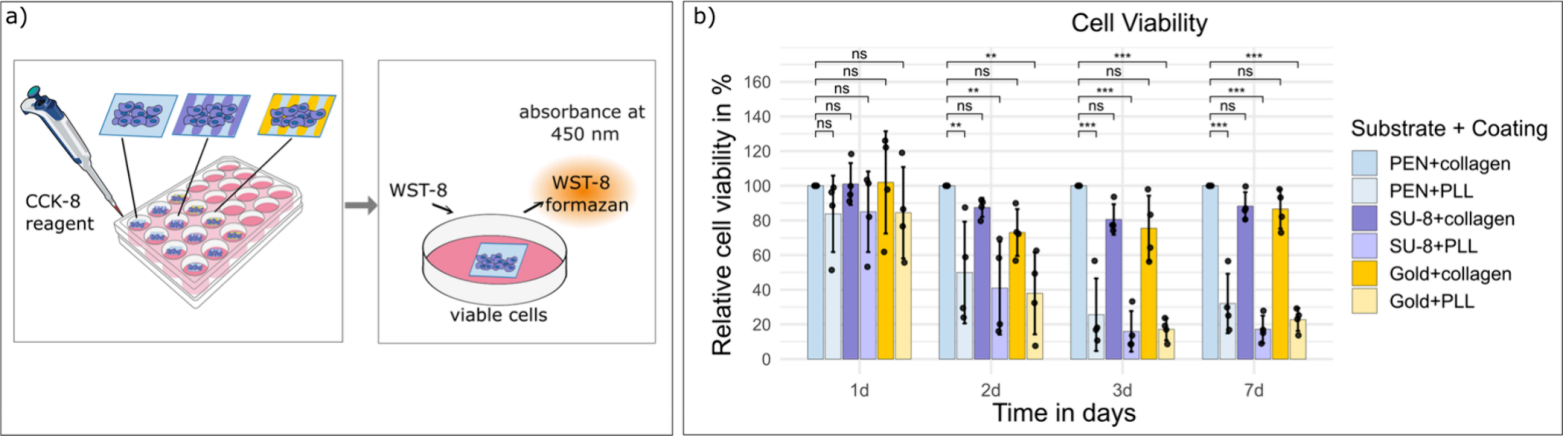
Confirmation of noncytotoxicity of printed inks on flexible foil. (a) Schematic of the cell counting kit-8 (CCK-8) cell viability assay workflow. Water-soluble tetrazolium salt-8 (WST-8) is metabolized by viable cells to produce a water-soluble formazan dye, which is quantified by absorbance at 450 nm. (b) Relative cell viability of HT-29 cells cultured for 1, 2, 3, and 7 days on polyethylene-naphthalate (PEN), SU-8 and gold surfaces coated with collagen or poly-l-lysine (PLL). Relative cell viability was calculated by normalizing to the collagen-coated and time-matched PEN reference. Data represent mean ± SD of 4 tested foils per substrate. Statistical comparisons were performed against the collagen-coated foil reference at each time point using one-way ANOVA with Dunnett’s post hoc test. ns, not significant. *, *p* < 0.05. **, *p* < 0.01. ***, *p* < 0.001.

## Conclusions

We realized a flexible microelectrode array through a fully additive, maskless four-step inkjet-printing process that defined both conductors and insulation. The printed MEAs were optically, electrically, and electrochemically characterized, revealing a rough gold morphology with an increased effective surface area. This resulted in reduced electrode impedance and significantly enhanced cathodic charge density compared to sputtered gold electrodes of similar size, enabling more efficient electrical stimulation.

We successfully recorded neuronal signals with SNRs exceeding 5 and achieved reliable neural activation through electrical stimulation. The suitability of the materials for cell interfacing was confirmed with a standard cell viability assay. Taken together, the results establish additively manufactured MEAs as a versatile and scalable technology that lowers the barrier of creating tailored neural interfaces.

This technology will have a significant impact on basic neuroscience, organ-on-chip systems, and wearable devices. Future work could involve extending the use of printed MEAs to three-dimensional tissue systems such as spheroids and organoids. While further optimization of electrode materials and long-term stability will be important for chronic applications, the demonstrated platform lays the groundwork for future neural interfaces that can be rapidly tailored to diverse biological models and experimental demands, supporting increasingly complex and application-specific bioelectronic systems. The additive noncontact printing approach allows fabrication on thin, sensitive, and nonplanar substrates, including membranes, meshes, and three-dimensional structures, beyond the limits of conventional photolithography.

## Experimental Section

### Design of the Printed Electrode Array

The digital bitmaps were created using Paint 2024, Inkscape 1.2, and AutoCAD 2023. During the printing process, each colored pixel in the bitmap is converted into a droplet that is then printed onto the substrate, thereby forming the electrode arrays and test structures. Electrode arrays were designed to fit into a commercial amplifier, the MEA2100-Mini Headstage (MultiChannel Systems), with their contact pads. To reduce stray capacitance in the final electrode array, feedlines in the middle of the electrode array, where they contact the liquid, were printed at approximately 50 μm wide, while broader gold lines were used as the outer feedlines. Dielectric ink was then printed on top of the feedlines to provide insulation, while keeping the electrode interface and contact pads open. Thus, all structures on the substrate were fabricated using an additive inkjet-printing process.

### Printing Technology and Ink Preparation

A commercial inkjet-printer, PixDro LP50 (Süss MicroTec), was equipped with Dimatix Materials Cartridge Samba with a drop volume of 2.4 pL. The cartridges have a piezoelectric drop-on-demand technology with a minimum dot size of 30 μm. These cartridges require an ink surface tension of 28–32 dyn cm^–1^ and a viscosity of 4–8 cPs for reliable jetting and printing results.^40^ The epoxy-based negative photoresist XP PriElex SU-8 1.0 (Kayaku Advanced Materials, USA) with a surface tension of 30 dyn cm^–1^ and a viscosity of 9.19 cSt was store-bought. To ensure printability, the ink was diluted using cyclopentanone (Sigma-Aldrich, Germany). A dilution with 8% cyclopentanone led to a viscosity of 8.5 cPs and 16 wt % to 6 cPs measured at room temperature (with a piezoelectric MEMS viscosity sensor^41^). A dilution of 11 wt % was chosen after showing the most reliable jetting while printing. The surface tension of the inks was measured with an OCA 15EC angle measurement device (DataPhysics Instruments GmbH, Germany). The 11% diluted SU-8 ink XP PriElex SU-8 1.0 will, from here on, be called SU-8. Commercially bought conductive ink was used as purchased. The voltage pulse controlling the piezoelectric drop-on-demand system was varied in terms of pulse width, voltage amplitude, and pulse distance. Other optimized printing parameters were printing speed and resolution in dpi, which defined the drop distance. One nozzle was used to ensure the correct alignment of lines in the micrometer range.

### Inkjet-Printing of Electrode Arrays

Printed electrode arrays on PEN had an outer dimension of 49.5 × 49.5 mm^2^. This size was selected to fit commercial MEA amplifiers. We inkjet-printed gold nanoparticle ink Drycure Au-J 0410 (C-ink, Japan)^14^ and SU-8 ink. As a substrate, TEONEX Q83 (PEN) foil with a thickness of 75 μm (Pütz GmbH + Co. Folien KG, Germany), glass slides with a thickness of 1 mm, and PMMA (POLYVANTIS GmbH, Germany) slides with a thickness of 1.5 mm were employed. Inks were filtered with a 0.45 μm hydrophilic PTFE filter to avoid clogging of the ⌀ 30 μm nozzles by unwanted particles. The printhead was operated under the following conditions: excitation voltage pulses controlling the piezo-driven printheads varied from 1.8 to 2.4 μs pulse length with a spacing from 2.5 to 4.9 μs between pulses, depending on the fluid properties inside the printhead. A 60 V μs^–1^ slew rate was determined for each voltage pulse, and an amplitude of 38–40 V. For SU-8, the printhead was heated to 45 °C, while it was not heated for the low-temperature curing gold ink, to prevent nozzle clogging (Table S2 and Figure S1). For all printing, the inkjet-printer was connected to an exhaust system and SU-8 was handled under the fume hood. After printing, the gold ink was cured at 80 °C for 1 h. SU-8 was soft-baked for 2 min, ramping from 65 to 95 °C, exposed to UV-Light for 2 min, and subsequently postexposure baked for 10 min at 110 °C. Chambers to hold liquid on top of the MEAs were fabricated with a DLP printer and Biomed Clear Resin, cured according to the postprocessing guidelines. Subsequently, they were glued with Silpuran 4200 biocompatible glue (QUAX GmbH, Germany).

### Characterization of Printed Structures

Electrode arrays were characterized using brightfield microscopy, AFM, SEM, and height profilometry using a Dektak XT profilometer. Electrode areas and pitch were determined with ImageJ analysis. The surface morphology of gold printed on PEN substrates was analyzed by AFM. Measurements were performed over a scanning area of 3 μm × 3 μm at a scanning speed of 0.6 μm s^–1^ with 1024 samples per line. The root-mean-square surface roughness was determined at three distinct locations within each recorded image. We performed profilometry measurements to determine the cross-sectional area of the fabricated structures. Each sample was measured three times using a stylus-based profilometer, and the extracted profile data were processed to compute the average cross-sectional area. Additionally, we conducted four-point probe resistance measurements using a wafer prober setup. For each sample, three resistance measurements were taken. Using the measured resistance values, the extracted cross-sectional areas, and the known line lengths (5, 10, and 15 mm), we calculated the specific resistance ρ of each structure. The final values were expressed in μΩ cm.

$$\rho =\frac{R\cdot A}{l}$$

The same printed electrode arrays were used for three independent recordings of action potentials and electrical stimulation in ex vivo mouse retinae over a period of 9 months, demonstrating overall device stability. Minor mechanical surface scratches of the thin printed gold contact pads were occasionally observed during handling, a known limitation of thin printed metal layers. These defects were readily repaired using silver paste (silver conductive epoxy adhesive 8331S, MG Chemicals), as also reported previously,^7^ without affecting device performance. Future work will focus on improving mechanical robustness, for example by increasing metal thickness, or reinforcing contact pad regions.

### Electrochemical Characterization and Measurement of Stimulation Currents

We conducted EIS to analyze our printed electrode arrays’ electrochemical properties. A round chamber was fixed on the electrode array and filled with PBS. Impedances were measured using a three-electrode setup, with a platinum counter electrode and an Ag/AgCl reference electrode. The Sciospec MEArack and ISX-3 Impedance Analyzer (Sciospec Scientific Instruments GmbH, Germany) were utilized with an excitation voltage of 100 mV and a frequency range of 10 Hz to 100 kHz.

Stimulation currents were measured using a DHPCA-100 (FEMTO, Germany) transimpedance amplifier. The working electrode on the printed electrode array was connected to a function generator controlled by a customized Matlab (Mathworks, USA) script. We recorded ten repetitions of voltages ranging from 0.1 to 1.9 V against an Ag/AgCl reference electrode for three electrodes for each MEA. The data was read out using an IFB-C board (Multi Channel Systems MCS GmbH, Germany). For comparison, these electrochemical characterization procedures were also obtained for a glass MEA with sputtered gold microelectrodes (Multi Channel Systems MCS GmbH, Germany). CV was conducted with a Palmsens 4 (PalmSens BV, Netherlands) with a scan rate of 0.1 V s^–1^.

### Cytotoxicity Testing

Gold ink and SU-8 ink were printed onto 9 × 9 mm^2^ sheets of PEN foil. SU-8 was prebaked from 65 to 110 °C for 2 min, exposed to UV light for 2 min, and postexposure baked at 110 °C for 10 min. Gold ink was cured for 1 h at 80 °C. Prior to cell seeding, the foils underwent plasma treatment at 80 W for 100 s using a Zepto Plasma Cleaner (Diener, Germany) to improve cell adhesion. Each foil was then placed in 24-well plates (Falcon) with untreated wells to minimize background attachment. Surfaces were cleaned and sterilized through sequential washes with 500 μL of 70% ethanol, a 30 min incubation in 500 μL of 70% ethanol, air-drying and 30 min of UV exposure, followed by two rinses with 1 mL of 1× phosphate-buffered saline (PBS; Gibco). To further facilitate adhesion, foils were coated overnight with either 250 μL of 10% Bovine Collagen Type I (Merck) or 0.01 μg μL^–1^ poly-l-lysine (PLL; MW 150–300 kDa, Sigma-Aldrich). The following day, coating solutions were removed and the foils were rinsed three times with 500 μL PBS and 500 μL of culture medium, making them ready for seeding.

HT-29 cells (ATCC), a human adherent epithelial line, were selected to provide an initial evaluation of potential epithelial or mucosal tissue exposure, which is relevant for assessing material interactions at barrier surfaces. The cells were maintained in high-glucose Dulbecco’s modified Eagle’s medium (DMEM; without l-glutamine or sodium pyruvate; Gibco) supplemented with 10% heat-inactivated fetal bovine serum (FBS; Gibco), 1% penicillin–streptomycin (Gibco) and 1% l-glutamine (Gibco) at 37 °C in 5% CO_2_. Cells were detached using trypsin (Gibco) and seeded onto the foils at a density of 1 × 10^5^ per well.

Cell viability was evaluated at 1, 2, 3, and 7 days using the colorimetric CCK-8 assay (MedChemExpress) according to the manufacturer’s instructions. Prior to measurement, the medium was removed and replaced with 300 μL of fresh medium to ensure that only attached cells contributed to the signal. 10% CCK-8 solution was added to each well and incubated for 2 h. Then, 100 μL of this solution was transferred to a 96-well plate (Thermo Fisher Scientific Inc.) to prevent interference from the substrate and absorbance was measured at 450 nm (Byonoy GmbH, Germany). Relative viability was calculated as cell viability (%) = (sample signal – blank signal)/(control signal – blank signal) × 100, where blanks consisted of CCK-8 in medium without cells. Because the assay permits continuous culture, the solution was removed after measurement, wells were washed with 500 μL PBS and 500 μL medium, and cells were returned to 500 μL of fresh medium for further incubation. Four foils per surface treatment were analyzed. Statistical comparisons to the collagen-coated PEN control were performed using one-way ANOVA with Dunnett’s post hoc test in R (v4.4.3).

### Extracellular Electrophysiology

Extracellular electrophysiology measurements were conducted using ex vivo mouse retinae. The experimental procedures for the preparation of ex vivo retinae were approved by the Center for Biomedical Research, Medical University of Vienna. All breeding and experimentation were performed under a license approved by the Austrian Federal Ministry of Science and Research in accordance with Austrian and EU animal laws (BMWFW-66.009/0403-WF/V/3b/2014).

For all experiments, rod-degenerated blind transgenic mice (79–129 days postnatal, rd10; B6.CXB1-Pde6brd10/J) were used. The printed MEAs were cleaned with 70% EtOH in distilled water, followed by distilled water, and subsequently plasma-cleaned at 80 W for 2 min using a Zepto Plasma Cleaner (Diener, Germany). The electrodes were coated with 200 μL poly-l-lysine (1 mg mL^–1^, P1399, MW 150–300 kDa, Sigma-Aldrich) before placing the retinal tissue to improve adhesion. During recordings, the MEAs were placed inside a commercial amplifier (Multi Channel Systems MCS GmbH, Germany) and perfused with carbogenated Ames’ medium (Sigma-Aldrich; 95% O_2_/5% CO_2_) heated to 32–35 °C at a flow rate of ∼3 mL min^–1^ using an inline heater (Multi Channel Systems GmbH, Germany). Signals were recorded at 50 kHz. Extracellular recordings with the printed MEAs were conducted epiretinally, i.e., with the retinal ganglion cell (RGC) layer contacting the printed electrodes. For electrical stimulation experiments using the flexMEA and patch clamp recordings, the retina was stimulated subretinally, i.e., with the photoreceptor layer contacting the printed electrodes.

Signals recorded from the printed electrodes were filtered using a second-order low-pass filter (5000 Hz) and a second-order high-pass filter (200 Hz). Spike sorting was performed by threshold-based spike detection, waveform extraction, feature extraction using principal component analysis (PCA), and clustering using the K-means Python library. The signal-to-noise ratio (SNR) was calculated as the mean peak-to-peak signal amplitude relative to the noise estimated from a 30 s trace after subtracting detected spikes.

For electrical stimulation experiments, the retina was sandwiched between the printed MEA and a perforated flexMEA with 32 electrodes (NMI TT, Germany). The flexMEA was lowered onto the sample using a micromanipulator (MPC-200, Sutter Instruments). Electrical stimulation was applied subretinally at the photoreceptor side using the printed MEA connected to a function generator, while recordings were performed epiretinally using the flexMEA. The flexMEA was connected via preamplifiers (Multi Channel Systems MCS GmbH, Germany) to the IFB-C board together with the trigger signals from the function generator. The signals were filtered, aligned to the recorded triggers, and stimulation artifacts were subtracted and zeroed. Spikes were detected using a threshold of five times the median noise level of each channel. The first 7 ms after stimulus onset were excluded from spike detection, and spikes occurring within the first 20 ms after stimulus onset were classified as stimulus-induced responses. The stimulation threshold was determined from 10 repetitions per voltage pulse amplitude at a probability of 66% for evoking a spike.

For patch clamp recordings, small holes were made in the inner limiting membrane to obtain access to retinal ganglion cell somata. Spiking responses were recorded using loose-patch recordings with a HEKA EPC-10 USB amplifier (HEKA, Multi Channel Systems MCS GmbH, Germany) at a sampling rate of 50 kHz. Similar to the flexMEA recordings, stimulation triggers were recorded to align stimulation and responses. Each stimulus amplitude was repeated 10 times, and responses were detected between 7 and 50 ms after stimulus onset.

We conducted extracellular electrophysiology measurements using an ex vivo mouse retina to validate the printed electrode array’s functionality. The experimental procedures for the preparation of ex vivo retinae were approved by the Center for Biomedical Research, Medical University of Vienna. All breeding and experimentation were performed under a license approved by the Austrian Federal Ministry of Science and Research in accordance with the Austrian and EU animal laws (BMWFW-66.009/0403-WF/V/3b/2014).

For recording with the printed MEAs, we used a rod-degenerated blind transgenic male mouse (rd10; B6.CXB1-Pde6brd10/J) 129 days postnatal. The printed MEAs were first cleaned with 70% EtOH in distilled water and with distilled water, and subsequently plasma cleaned at 80 W for 2 min with a Zepto Plasma Cleaner (Diener, Germany). We coated the electrodes with 200 μL poly-l-lysine (1 mg mL^–1^, P1399, MW 150–300 kDa, Sigma-Aldrich) before placing the retinal tissue to improve adhesion. The printed MEA was placed inside a commercial Amplifier (Multi Channel Systems MCS GmbH, Germany) and the chamber was perfused with carbogenated and heated (32–35 °C) Ames’ medium (Sigma-Aldrich; 95% O_2_/5%CO_2_) at a flow rate of ∼3 mL min^–1^ with an inline heater (Multi Channel Systems GmbH, Germany). We recorded at 50 kHz using the MultiChannel Experimenter software. The measurements of extracellular action potentials were filtered with a second-order low-pass filter (5000 Hz) and a second-order high-pass filter (200 Hz). Cell sorting was done by threshold-based detection of the spikes, extraction of the waveforms, feature extraction with Principal Component Analysis (PCA), and clustering of the features with the Kmeans Python library. The SNR was calculated with the peak-to-peak signal amplitude mean vs the noise calculated from a 30 s trace with the peaks subtracted.

For MEA recordings in combination with electrical stimulation, we used a rod-degenerated blind transgenic female mouse (rd10; B6.CXB1-Pde6brd10/J) 118 days postnatal. In this setup, we sandwiched the retina between the printed MEA and a perforated flexMEA with 32 electrodes^28^ (NMI TT, Germany). The tissue was placed on the printed MEA, and the flex MEA was lowered onto the sample with a micromanipulator (MPC 200, Sutter Instruments). We stimulated subretinally at the side of the photoreceptors with the printed MEA connected to a function generator, while recording epiretinally at the side of the optic nerve with the flexMEA. The flexMEA was connected over preamplifiers (Multi Channel Systems MCS GmbH, Germany) to the IFB-C Board, as well as the triggers from the function generator. To analyze the cell response to the electrical stimulation, the signal was filtered, aligned with the recorded triggers, the stimulation artifacts were subtracted and zeroed and spikes were detected using 5 times the median noise of each channel as a threshold. The first 7 s after stimulus onset were not considered for spike detection, and spikes occurring in the first 20 ms after stimulus onset were counted as stimulus-induced responses. The stimulation threshold was determined from 10 repetitions per voltage pulse amplitude at a 66% probability of evoking a spike after the stimulus.

## Supplementary Material



## Notes

Additional information on SU-8 optimization on SU-8 optimization and ink jetting; impedance data and microscopic images showing low device-to-device variability; long-term recordings with ex vivo mouse retinae further demonstrate robust device performance; and detailed printing parameters (PDF)

## References

[1] Obien M. E. J., Deligkaris K., Bullmann T., Bakkum D. J., Frey U. (2015). Revealing
Neuronal Function through Microelectrode Array Recordings. Frontiers in Neuroscience.

[2] Williams N. P., Voroslakos M., Shi D., Pwint M. Y., Lanzio V., Mao H., Zolotavin P., Yoon E., Stieglitz T., Xie C., Harris T. D., Schwartz A. B., Cui X. T. (2025). In Vivo Microelectrode
Arrays for Neuroscience. Nature Reviews Methods
Primers.

[3] Hassler C., Boretius T., Stieglitz T. (2011). Polymers for Neural Implants. Journal of Polymer Science, Part B: Polymer Physics..

[4] Adly N., Teshima T. F., Hassani H., Boustani G. Al, Weiß L. K., Cheng G., Alexander J., Wolfrum B. (2023). Printed Silk Microelectrode
Arrays for Electrophysiological Recoding and Controlled Drug Delivery. Adv. Healthc. Mater..

[5] Bachmann B., Adly N. Y., Schnitker J., Yakushenko A., Rinklin P., Offenhäusser A., Wolfrum B. (2017). All-Inkjet-Printed
Gold Microelectrode Arrays for Extracellular Recording of Action Potentials. Flexible and Printed Electronics.

[6] Garma L. D., Ferrari L. M., Scognamiglio P., Greco F., Santoro F. (2019). Inkjet-Printed
PEDOT:PSS Multi-Electrode Arrays for Low-Cost in Vitro Electrophysiology. Lab Chip.

[7] Lumpuy-Castillo J., Fu Y., Avila Ramirez A. E., Solodka K., Li J., Lorenzo O., Zeglio E., Garma L. D. (2025). Inkjet-Printed Graphene
Multielectrode Arrays: An Accessible Platform for In Vitro Cardiac
Electrophysiology. ACS Appl. Bio Mater..

[8] Kopic I., Peng H., Schmidt S., Berezin O., Wang S., Westmeyer G. G., Wolfrum B. (2025). Inkjet-Printed 3D Sensor Arrays with
FIB-Induced Electrode Refinement for Low-Noise Amperometric Recordings
in HiPSC-Derived Brain Organoids. ACS Sens..

[9] Kopic I., Dedousi P., Schmidt S., Peng H., Berezin O., Weiße A., George R. M., Mayr C., Westmeyer G. G., Wolfrum B. (2024). Inkjet-Printed 3D Electrode Arrays for Recording Signals
from Cortical Organoids. Adv. Mater. Technol..

[10] Grob L., Rinklin P., Zips S., Mayer D., Weidlich S., Terkan K., Weiß L. J. K., Adly N., Offenhäusser A., Wolfrum B. (2021). Inkjet-Printed and
Electroplated 3D Electrodes for
Recording Extracellular Signals in Cell Culture. Sensors.

[11] Vėbraitė I., Bar-Haim C., David-Pur M., Hanein Y. (2024). Bi-Directional Electrical
Recording and Stimulation of the Intact Retina with a Screen-Printed
Soft Probe: A Feasibility Study. Front. Neurosci..

[12] Borda E., Ferlauto L., Schleuniger J., Mustaccio A., Lütolf F., Lücke A., Fricke S., Marjanović N., Ghezzi D. (2020). All-Printed Electrocorticography
Array for In Vivo
Neural Recordings. Adv. Eng. Mater..

[13] Kokubo N., Arake M., Yamagishi K., Morimoto Y., Takeoka S., Ohta H., Fujie T. (2019). Inkjet-Printed
Neural Electrodes
with Mechanically Gradient Structure. ACS Appl.
Bio Mater..

[14] Sharova A. S., Modena F., Luzio A., Melloni F., Cataldi P., Viola F. A., Lamanna L., Zorn N. F., Sassi M., Ronchi C., Zaumseil J., Beverina L., Antognazza M. R., Caironi M. (2023). Chitosan Gated Organic Transistors Printed on Ethyl
Cellulose as a Versatile Platform for Edible Electronics and Bioelectronics. Nanoscale.

[15] Cogan S. F. (2008). Neural
Stimulation and Recording Electrodes. Annu.
Rev. Biomed. Eng..

[16] Boehler C., Carli S., Fadiga L., Stieglitz T., Asplund M. (2020). Tutorial: Guidelines for Standardized
Performance Tests
for Electrodes Intended for Neural Interfaces and Bioelectronics. Nature Protocols.

[17] Fan B., Wolfrum B., Robinson J. T. (2021). Impedance
Scaling for Gold and Platinum
Microelectrodes. J. Neural Eng..

[18] Cui H., Xie X., Xu S., Chan L. L. H., Hu Y. (2019). Electrochemical
Characteristics
of Microelectrode Designed for Electrical Stimulation. Biomed. Eng. Online.

[19] Zeck G., Jetter F., Channappa L., Bertotti G., Thewes R. (2017). Electrical
Imaging: Investigating Cellular Function at High Resolution. Adv. Biosyst..

[20] Reinhard K., Tikidji-Hamburyan A., Seitter H., Idrees S., Mutter M., Benkner B., Münch T. A. (2014). Step-By-Step Instructions for Retina
Recordings with Perforated Multi Electrode Arrays. PLoS One.

[21] Corna A., Ramesh P., Jetter F., Lee M.-J., Macke J. H., Zeck G. (2021). Discrimination of Simple Objects
Decoded from the Output of Retinal
Ganglion Cells upon Sinusoidal Electrical Stimulation. J. Neural Eng..

[22] Corna A., Cojocaru A. E., Bui M. T., Werginz P., Zeck G. (2024). Avoidance
of Axonal Stimulation with Sinusoidal Epiretinal Stimulation. J. Neural Eng..

[23] Buccino A. P., Hurwitz C. L., Garcia S., Magland J., Siegle J. H., Hurwitz R., Hennig M. H. (2020). Spikeinterface,
a Unified Framework
for Spike Sorting. Elife.

[24] Wang Q., Song S., Wang W., Zhou J., Riaud A. (2022). On the Dynamic
Stability of Gold Electrodes Exposed to Alternative Voltages in Microfluidic
Systems. J. Electrochem. Soc..

[25] Bhalla V., Carrara S., Stagni C., Samorì B. (2010). Chip Cleaning
and Regeneration for Electrochemical Sensor Arrays. Thin Solid Films.

[26] Agüí L., Peña L., Pedrero M., Yáñez-Sedeño P., Pingarrón J. M. (2002). Determination of Disulfiram by Adsorptive Stripping
Voltammetry at Gold Disk Microelectrodes. Electroanalysis.

[27] Ganji M., Tanaka A., Gilja V., Halgren E., Dayeh S. A. (2017). Scaling
Effects on the Electrochemical Stimulation Performance of Au, Pt,
and PEDOT:PSS Electrocorticography Arrays. Adv.
Funct. Mater..

[28] Corna A., Herrmann T., Zeck G. (2018). Electrode-Size Dependent Thresholds
in Subretinal Neuroprosthetic Stimulation. J.
Neural Eng..

[29] Eickenscheidt M., Jenkner M., Thewes R., Fromherz P., Zeck G. (2012). Electrical
Stimulation of Retinal Neurons in Epiretinal and Subretinal Configuration
Using a Multicapacitor Array. J. Neurophysiol..

[30] Boinagrov D., Pangratz-Fuehrer S., Goetz G., Palanker D. (2014). Selectivity of Direct
and Network-Mediated Stimulation of the Retinal Ganglion Cells with
Epi-, Sub- and Intraretinal Electrodes. J. Neural
Eng..

[31] Ho E., Smith R., Goetz G., Lei I., Galambos L., Kamins T. I., Harris J., Mathieson K., Palanker D., Sher A. (2018). Spatiotemporal Characteristics of
Retinal Response to Network-Mediated Photovoltaic Stimulation. J. Neurophysiol..

[32] Sekirnjak C., Hottowy P., Sher A., Dabrowski W., Litke A. M., Chichilnisky E. J. (2006). Electrical
Stimulation of Mammalian
Retinal Ganglion Cells with Multielectrode Arrays. J. Neurophysiol..

[33] Ho E., Lorach H., Goetz G., Laszlo F., Lei X., Kamins T., Mariani J. C., Sher A., Palanker D. (2018). Temporal Structure
in Spiking Patterns of Ganglion Cells Defines Perceptual Thresholds
in Rodents with Subretinal Prosthesis. Scientific
Reports.

[34] Corna A., Gugujonovic K., Schiek M., Bellapianta A., Werginz P., Ziller A., Scharber M. C., Salti A., Bolz M., Irimia-Vladu M., Zeck G. (2026). Near-Infrared Organic
Photovoltaic Electrodes for Subretinal Neurostimulation. Advanced Functional Materials.

[35] Pranti A. S., Schander A., Bödecker A., Lang W. (2018). PEDOT: PSS Coating
on Gold Microelectrodes with Excellent Stability and High Charge Injection
Capacity for Chronic Neural Interfaces. Sens.
Actuators B Chem..

[36] Samba R., Herrmann T., Zeck G. (2015). PEDOT-CNT Coated Electrodes
Stimulate
Retinal Neurons at Low Voltage Amplitudes and Low Charge Densities. J. Neural Eng..

[37] Tominaga H., Ishiyama M., Ohseto F., Sasamoto K., Hamamoto T., Suzuki K., Watanabe M. (1999). A Water-Soluble Tetrazolium
Salt Useful
for Colorimetric Cell Viability Assay..

[38] Naveen K. V., Tyagi A., Ibrahium O. M. H., Fischer R. E. A. W., Ostafe R. (2026). From Dye Exclusion to High-Throughput Screening: A
Review of Cell Viability Assays and Their Applications. Biotechnol. Adv..

[39] Ell M., Bui M. T., Kigili S., Zeck G., Prado-López S. (2024). Assessment
of Chemotherapeutic Effects on Cancer Cells Using Adhesion Noise Spectroscopy. Front. Bioeng. Biotechnol..

[40] FUJIFILM Dimatix, Inc. Dimatix® Materials Cartridge—Samba® Cartridge. https://asset.fujifilm.com/www/mx/files/2021-04/ae8a1e167ce8c273fcdd31ecffd9ec80/PDS00142.pdf (accessed Dec 09, 2025).

[41] Alasatri S., Schneider M., Mirwald J., Hofko B., Schmid U. (2022). Accuracy and
Precision of Resonant Piezoelectric MEMS Viscosity Sensors in Highly
Viscous Bituminous Materials. Sens. Actuators
A Phys..

