# Supplementary material for: Inkjet-Printed Rough Gold Microelectrode Arrays on Flexible Substrates for Neural Recording and Electrical Stimulation
Source: ACS Appl Mater Interfaces. 2026 May 4;18(18):25835–46. doi: 10.1021/acsami.6c01300 (PMC13181718; doi:10.1021/acsami.6c01300)
Supplement: Supplementary file 1 [file am6c01300_si_001.pdf]

Supporting Information

Inkjet-printed Rough Gold Microelectrode Arrays on Flexible Substrates for Neural Recording and Electrical Stimulation

*Authors: Amelie Ziller [a], Andrea Corna [a], Mai Thu Bui [a], Paul Werginz [a], Michael Schneider [b], Ulrich Schmid [b], Günther Zeck [a]**

[a] Institute of Biomedical Electronics, TU Wien, Gußhausstraße 27-29, 1040 Vienna

[b] Institute of Sensor and Actuator Systems, TU Wien, Gußhausstraße 27-29, 1040 Vienna

Email: guenther.zeck@tuwien.ac.at

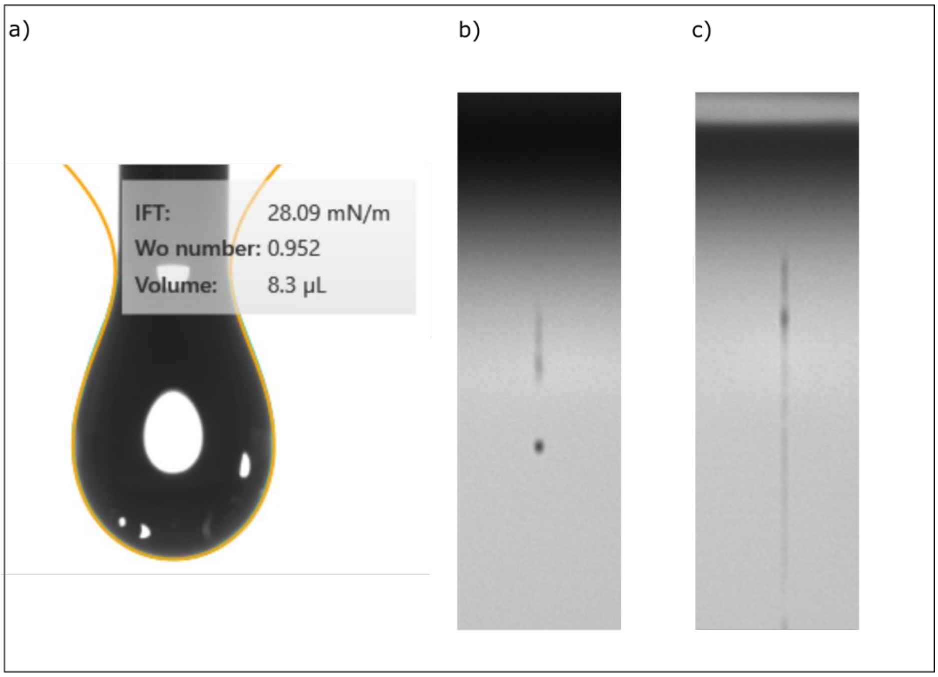

Supplementary Figure 1. SU-8 optimization and ink jetting. a) SU-8 ink optimization and characterization via surface tension. b) dropview image of SU-8 jetting and of c) gold jetting.

Parameters	Device 1<br>⌀ 51 µm ± 12 µm<br>(n = 18)	Device 2<br>⌀ 65 µm ± 7 µm (n = 30)	Device 3<br>⌀ 79 µm ± 8 µm (n = 32)	glass MEA<br>⌀ 40 µm<br>(n = 16)	glass MEA<br>⌀ 110 µm<br>(n = 7)
|Z| @ 1 kHz	41 kΩ	26 kΩ	19 kΩ	558 kΩ	141 kΩ
Phase @ 1 kHz	-53°	-49 °	-50 °	- 74°	-80.5
f_cutoff (-45°)	3.1 kHz	2.1 kHz	1.8 kHz	393.3 kHz	41.0 kHz

Supplementary Table 1. Device to device variability of impedance magnitude at 1 kHz, phase at 1 kHz and cutoff frequency at a phase of -45° of printed MEAs and sputtered gold microelectrodes for n electrodes.

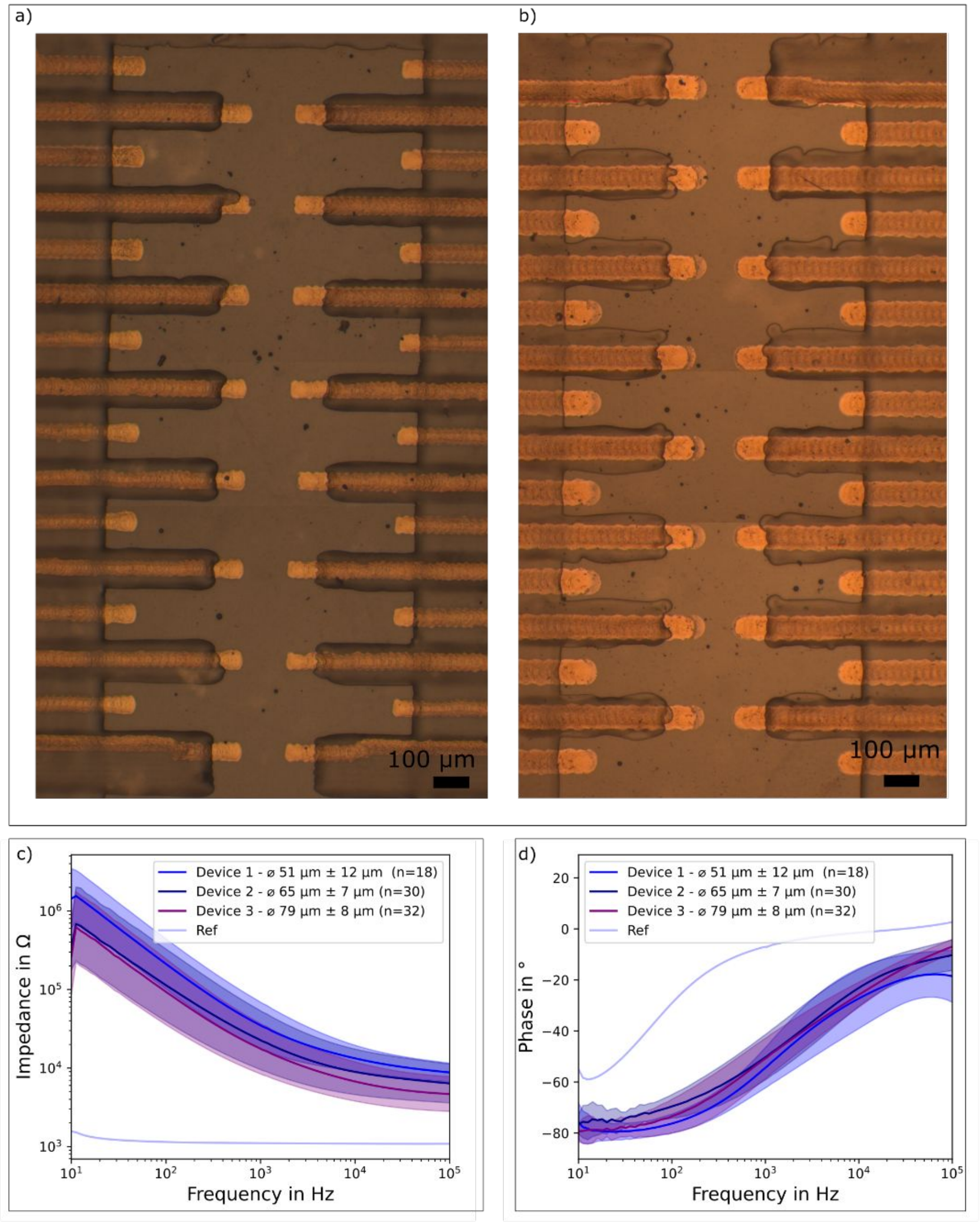

Supplementary Figure 2. Device to device variability. Optical microscopy images of additional microelectrode arrays. a) Device 2 and b) Device 3. Optimization of the plasma treatment (increased from 12 s to 20 s) was performed to ensure feedline continuity, resulting in the observed variance in electrode diameters (65 µm ± 7 µm vs. 79 µm ± 8 µm). c) average

impedance magnitude and standard deviation of Device 1, 2 and 3 and d) phase and standard deviation for n electrodes.

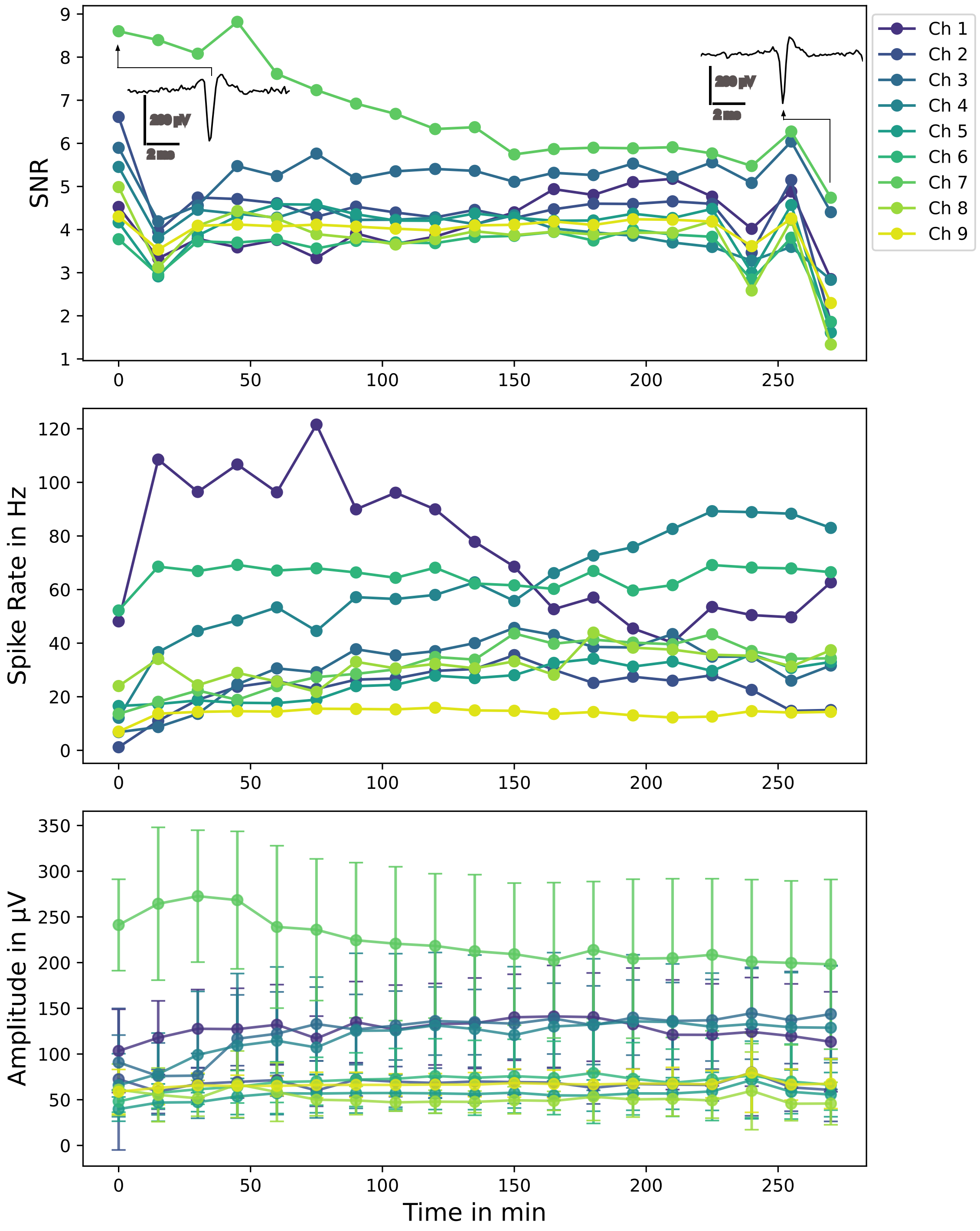

Supplementary Figure 3. **Long-term recording of the ex vivo retina.** Extracellular voltages were recorded for 2 minutes with a pause of 13 minutes between recordings, starting from 30 minutes after placement. Shown are the signal-to-noise ratios (upper graph), the spike rates

(middle graph) and the mean spike amplitudes with standard deviations (lower graph) of different electrodes. Each symbol denotes data from one electrode (“Channel 1 to 9”).

Ink	Print Speed	Piezoelectric Voltage pulse	Surface pretreatment
Gold	300 mm/s	37 V, 1.8 µs pulse width, 4.9 µs spacing, 60 V/µs slew rate	Cleaning with 70 % EtOH in distilled water and distilled water, Plasma treatment 12s, 80 W
SU-8	300 mm/s	40 V, 2.4 µs pulse width, 2.8 µs spacing, 60 V/µs slew rate	Air pressure cleaning to remove dust

Supplementary Table 2. Detailed print parameters for the PixDro LP50 with a Dimatix Materials Samba Cartridge with a drop volume of 2.4 pl for gold and SU-8 ink.
